# Total color change (Δ*E∗)* is a poor estimator of total carotenoids lost during post-harvest storage of biofortified maize grains

**DOI:** 10.1016/j.heliyon.2020.e05173

**Published:** 2020-10-06

**Authors:** Smith G. Nkhata

**Affiliations:** Agrofood Processing Technology, Faculty of Life Sciences and Natural Resources, Natural Resources College, Lilongwe University of Agriculture and Natural Resources, P.O Box 143, Lilongwe, Malawi

**Keywords:** Food science, Food analysis, PICS bags, Carotenoid degradation, Biofortified maize, Hue, Color parameters

## Abstract

Provitamin A biofortified maize is promoted in developing country to curb vitamin A deficiency. To determine the provitamin A carotenoid content of fresh and stored biofortified maize requires analytical techniques that are affordable by the targeted population. In this study color parameters (*L∗, a∗, b∗*) individually or in combination were used to estimate carotenoid content in high carotenoid biofortified maize. There was an increase in *L∗* value with storage indicating grains were becoming lighter while *a∗* and *b∗* values did not change significantly. Almost all storage bags induced total color change (Δ*E*∗) greater than 2 which is perceivable by consumers as a deviation from original quality. The coefficient of determination (R^2^) between carotenoid content and color parameters were high and significant for most color parameters suggesting that they could be used to estimate carotenoid content in biofortified maize. While change of color is indicative of carotenoid degradation, our study found that Δ*E*∗ is a poor estimator of carotenoids lost during post-harvest storage of biofortified maize. Hue (*h*∗), *L∗* and *a∗* gave consistently and significantly higher R^2^ (p < 0.05) for almost all carotenoids analyzed suggesting that they could be used to generate predictive models for estimating carotenoid content in stored biofortified maize.

## 1 Introduction

Change of color is indicative of carotenoid degradation in carotenoid rich food (Onwude et al., 2017). Carotenoids are organic pigments synthesized by plants and are responsible for red, orange and yellow colors in fruits and vegetables. They are highly unsaturated compounds that are prone to oxidation and isomerization mainly initiated by heat, light and oxygen that lead to change of color in food. When the carotenoids degrade the CIELAB color parameters that describe degree of lightness (*L∗*), redness (*a∗*) and yellowness (*b∗)* also change (Ganje et al., 2018). Despite having more than 650 species of carotenoids in nature, very few are found in human diets (Li et al., 2007). The commonest dietary carotenoids are lycopene, lutein, zeaxanthin, α-carotene, β-carotene and β-cryptoxanthin (Li et al., 2007). Carotenoids are associated with health benefits in humans with some studies reporting anti-degenerative properties as well as provitamin A activities (Johnson, 2014; Tang and Russell, 2009; Toti et al., 2018). Some carotenoids such as α-carotene, β-carotene and β-cryptoxanthin have vitamin A activity prompting promotion of biofortified orange maize and orange-fleshed sweet potatoes for consumption in developing countries in order to curb vitamin A deficiency (Biesalski et al., 2007).

A highly unsaturated nature of carotenoids renders them unstable during both processing and storage (Ganje et al., 2018). A significant loss in carotenoids was reported during post-harvest storage of high carotenoid biofortified orange maize (Nkhata et al., 2019; Taleon et al., 2017; Ortiz et al., 2016). Such losses significantly reduce both antioxidant and vitamin A activities of the grains making them lose their health promoting properties. Efforts to find a better storage technique that will maintain the nutritional quality of biofortified maize are underway and few studies have reported use of PICS bags as a promising strategy to reduce carotenoid loss during post-harvest storage (Nkhata et al., 2019; Taleon et al., 2017).

Degradation of carotenoids is detected visually using chromameter (colorimeter) or liquid chromatography (LC). Use of colorimeter to determine carotenoid content is relatively cheaper as does not require expensive equipment characteristic of LC. Biofortified maize is promoted in developing countries where availability of LC is limited necessitating the need for cheaper, rapid and non-destructive way of estimating carotenoid content in both fresh and stored grains. Use of less expensive chromameters offers an alternative to using LC in estimating carotenoid contents in grains. Different studies have found highly significant correlation between carotenoid content and color parameters in different food samples (Onwude et al., 2017; Arias et al., 2002; Melendez-Martinez et al., 2003; Andreu-Sevilla et al., 2008; Ruiz et al., 2008; Itle and Kabelka, 2009; Amenya and Wilson, 1997) suggesting that use of color parameters could be useful in estimating carotenoid content in maize grains especially in resource poor settings targeted for biofortification.

Different model kinetics have been developed though their goodness of fit is dependent on type of food product and carotenoid species (Onwude et al., 2017). These models include zero order (Onwude et al., 2017; Ruiz et al., 2008), first order (Onwude et al., 2017) exponential regression (Ruiz et al., 2008) and fractional conversion models (Onwude et al., 2017). It is clear from these models that none can be used to precisely estimate carotenoid content in all food samples highlight the complexity and challenge to use these models in different foods. Moreover, most of these models were developed in food samples that have one or two dominant carotenoids such as tomatoes (lycopene) (Ruiz et al., 2008; Ganje et al., 2018), pumpkin (β-carotene) (Onwude et al., 2017), sweet potato (β-carotene) (Amenya and Wilson, 1997), cashew nuts (β-carotene and β-cryptoxanthin) (Zepka et al., 2009), and therefore may be best suited to estimate carotenoids in those foods only. In a more complex food system such as maize grains where different carotenoids are in significant and comparable quantities, use of such models may not provide accurate estimates of carotenoids. Moreover the correlation of color parameters with different carotenoid contents had been shown to be different for different foods. For example *b∗* values correlated highly and significantly with β-carotene in white-freshed African sweet potato (Amenya and Wilson, 1997) while β-carotene and provitamin A activity was highly correlated with *a∗* value in peels of apricot (Itle and Kabelka, 2009). The *a∗* value had the best correlation with β-carotene content in orange and yellow flesh sweet potatoes (Takahata et al., 1993). *a∗* x *b∗* values were highly correlated with total carotenoids in papaya puree (Ahmed et al., 2002). As loss of color is a sign of carotenoid loss, in this study we tested whether total color change could be a reliable predictor of carotenoid lost in biofortified maize grain. Therefore the aim of this study was to determine changes in color parameters in relation to changes in carotenoid content during postharvest storage and identify the color parameters that could be used to quantitatively estimate carotenoid content in stored biofortified maize grains.

## 2 Materials and methods

### 2.1 Standards and solvents

Acetone, ethyl acetate, methanol (J. T. Baker, Phillipsburg, NJ, USA), methyl *tert*-butyl ether (MTBE) (Sigma-Aldrich, St. Louis, MO, USA) were all certified HPLC grade with >99.9% purity. A 1.0 M ammonium acetate solution for chromatography was prepared using double distilled water and adjusted to pH 4.6 with glacial acetic acid. Authentic carotenoid standards including lutein, *ß*-carotene, *ß*-cryptoxanthin, *ß*-*apo*-8′-carotenal (Sigma-Aldrich), zeaxanthin (IndoFine, Hillsborough, NJ, USA), *α*-carotene (CaroteneNature, Lupsingen, Switzerland) were obtained.

### 2.2 Packaging, storage and sampling of maize

Detailed description about storage, packing and sampling of maize have been reported previously (Nkhata et al., 2019). Briefly, two biofortified orange maize genotypes, open pollinated variety 1 (OPVI) and open-pollinated variety 2 (OPVII), were harvested and dried to ~8% moisture and then packed in PICS bags with oxygen scavengers enclosed (PICS-oxy), PICS bags without oxygen scavengers (PICS-noxy) and polypropylene woven bag (Woven) for 8 months. All bags were stored under same conditions; 29 °C and 30% rh. Sampling was done at 0, 0.5, 2, 4 and 8 months. At each sampling point representative grains were taken from each bag and stored at -80 °C. Color measurement and milling was done within one week after sampling.

### 2.3 Carotenoid extraction

Maize carotenoids were extracted as previously reported (Nkhata et al., 2019). Briefly, **~**500 mg of milled grain samples was spiked with 100 *μ*l of *ß-apo-8*-carotenal as standard and then extracted twice with 5 mL of chilled acetone and then extracted again twice with 2 mL of MTBE. Following extractions, MTBE fraction and acetone fraction were combined and dried under stream of nitrogen gas. Prior to LC analysis, dried carotenoids were solubilized in 2 mL of 1:1 ethyl acetate:methanol and filtered through 0.45*μ*m polytetrafluoroethylene (PTFE) filter and analyzed immediately by LC. Extraction recovery of this method was determined from recovery of the internal standard and was found to be 95.3 ± 3.6%.

### 2.4 LC analysis

Carotenoid separation was carried out on YMC C30 3 μm 2.0 mm × 150 mm column, with a YMC carotenoid guard column (2.0 × 23 mm) (YMC, Allentown, PA, USA) in a HP 1090 HPLC equipped with a Diode Array Detector scanning at 450 nm. Samples were eluted at 0.37 mL/min under the gradient conditions described previously (Kean et al., 2008). Carotenoid peaks were identified by co-chromatography with authentic *all-trans*-carotenoid standards and comparison with spectral information from literature as described previously (Kean et al., 2008). Quantitation was completed using a seven point response curve constructed with authentic carotenoid standards in the range of 0.01–8.0 μM.

### 2.5 Color analysis

Color measurements were taken at each testing interval on the maize grains using chromameter tristimulus color analyzer (CR-400 Series, Konica, Minolta Optics Inc, Japan) calibrated with a white porcelain reference plate. The chromameter took 5 readings in succession and generated an average number for each sample. Color parameters were quantified using CIELAB parameters (*L∗, a∗, b∗*) generated by chromameter. Total color change *(*Δ*E∗),* chroma (*C∗*) and hue (*h*) were calculated using the formulae; Δ*E∗* = $\sqrt{{\left(\text{Δ}L*\right)}^{2}+{\left(\text{Δ}a*\right)}^{2}+{\left(\text{Δ}b*\right)}^{2}}$, where Δ*L∗*, Δ*a∗* and Δ*b∗* represent changes in lightness, redness and yellowness, respectively, after specified period of time (month)*; C∗* = $\sqrt{{\left(a*\right)}^{2}+{\left(b*\right)}^{2}}$ , where *a∗* and *b∗* represent *a∗* value and *b∗* value after a specified period of time (month); and *h* = arctan$\left(\frac{b*}{a*}\right)$, where *b∗* and *a∗* represent *a∗* value and *b∗* values, respectively, after a specified time (month) (Ganje et al., 2018; Baik and Mittal, 2003). Associations between different parameters were determined using coefficient of determination (*R*^*2*^).

### 2.6 Data analysis

Data were analyzed by running ANOVA on SAS 94 version (SAS Institute Inc, NC) to generate treatment mean ± SE and coefficient of determination (*R*^*2*^) between carotenoid content and color parameters for maize stored in PICS-oxy, PICS-noxy and woven bags. Mean values for *L∗, a∗* and *b∗* for each storage system at each testing interval were calculated. Means were significantly different when p < 0.05 using Tukey's HSD test. The model *R*^*2*^ was significant when p < 0.05.

## 3 Results and discussions

### 3.1 Change in color parameters during storage of grains

**Figure 1:**
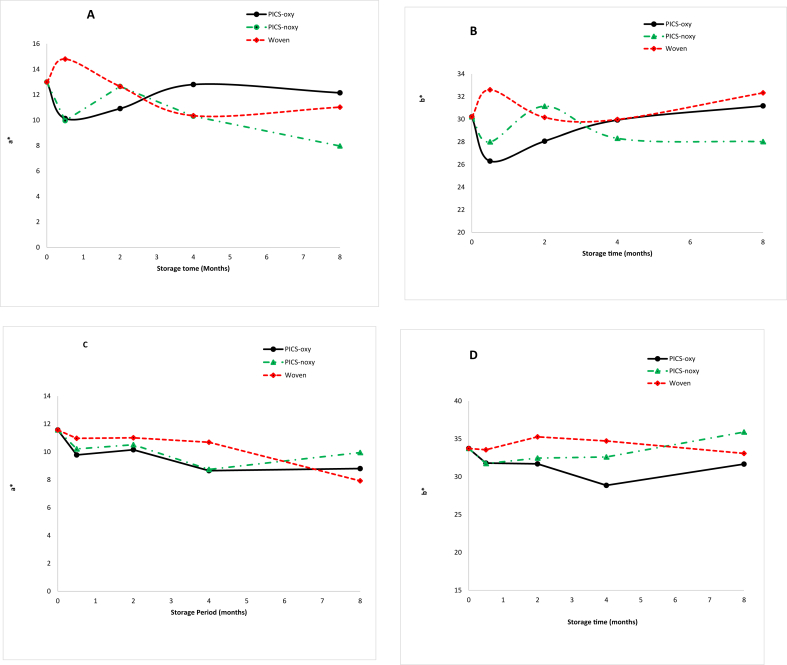
Changes in color parameters *a∗* and *b*∗ during 8 month storage in PICS-oxy, PICS-noxy and woven bags for OPVI (A, B) and OPVII (C, D). Each data point is an average of 4 determination (*n* = 4).

Carotenoid contents (*μ*g/g dry weight) of OPVI and OPVII used to compute coefficients of determination have been reported elsewhere (Nkhata et al., 2019). The changes in CIELAB color parameters (*L∗, a∗, b∗****,*** ΔE∗ and Chroma) are shown in Tables 1, 2, and 3 and Figure 1. *L∗* value generally increased after 8 month storage for both genotypes. The increase in *L∗* value suggests the grains were becoming lighter with storage and was associated with loss of carotenoids. There was no significant change in *a∗* and *b∗* values for both genotypes (Figure 1). *a∗* measures redness with higher positive values indicating redder color and higher negative value indicating greener color while *b∗* measures yellowness with higher positive values indicate a more yellow color and higher negative values indicate a more blue color (Qian et al., 2012; Ganje et al., 2018). Changes in Δ*E∗* and *C∗* did not follow a clearly defined pattern (Table 3). The magnitude of change in color parameters is not consistent with the magnitude of change in carotenoid contents recorded after 8 month storage (Nkhata et al., 2019) suggesting that decrease in carotenoid content in grains does not always produce proportionate changes in color parameters. Maize grains contain different carotenoids with different color intensities; therefore, it is possible that a decrease in one color parameter may result into an increase in a different color parameter during storage period which may result in an insignificant net color change despite a significant carotenoid loss. Use of tristimulus color parameters to estimate carotenoid content has been previously reported in tomatoes (Arias et al., 2002), orange juice (Melendez-Martinez et al., 2003), red palm oil (Andreu-Sevilla et al., 2008), apricot (Ruiz et al., 2008), pumpkin (Itle and Kabelka, 2009), sweet potatoes (Amenya and Wilson, 1997) and tomato paste (Ganje et al., 2018). The decrease in *L∗* was shown to be indicative of *β*-carotene and *β*-cryptoxanthin degradation during heating of cashew apple juice at 60 °C and 90 °C for 540 min and 240 min, respectively (Zepka et al., 2009). *L∗* value was also used to study degradation of β-carotene in β-carotene-enriched nanoemulsion during storage at different temperatures (Qian et al., 2012). The increase in *L∗* value in this study should not be confused with a decrease in *L∗* value reported in some studies where carotenoid degradation was induced by heating (Onwude et al., 2017; Zepka et al., 2009). Under such conditions the decrease in *L∗* values is due to the darkening of samples due to Maillard browning reaction (Onwude et al., 2017; Baik and Mittal, 2003) which is unlikely event in intact grains used in this study. As carotenoids degrade color intensity decreases while lightness (*L∗*) increases which is consistent with our results.

**Table 1:** Changes in lightness (*L∗*) during 8 month storage of OPVI in different storage bags.

Storage period (months)	PICS-oxy	PICS-noxy	Woven
0	62.3 ± 1.13a	62.3 ± 1.13b	62.3 ± 1.13ab
0.5	59.71 ± 1.96a	62.22 ± 0.73b	62.11 ± 0.63ab
1	64.67 ± 0.68a	63.11 ± 1.10ab	63.95 ± 0.63a
2	60.97 ± 2.29a	62.10 ± 1.11ab	57.64 ± 1.23b
4	62.26 ± 1.13a	63.38 ± 1.85ab	64.32 ± 1.20a
8	63.71 ± 1.66a	66.67 ± 1.30a	65.17 ± 1.40a

Means ± SE with different letters within a column are significantly different Tukey's test, α = 0.05. Each data point is an average of 4 determination (*n* = 4).

**Table 2:** Changes in lightness (*L∗*) during 8 month storage of OPVII in different storage bags.

Storage period (months)	PICS-oxy	PICS-noxy	Woven
0	61.39 ± 0.66b	61.39 ± 0.66b	61.39 ± 0.66c
0.5	63.46 ± 1.40b	63.11 ± 0.67ab	64.65 ± 1.47b
1	61.91 ± 1.75ab	62.31 ± 0.83ab	62.65 ± 0.88cb
2	65.05 ± 1.63ab	62.11 ± 0.63ab	64.08 ± 0.16b
4	65.69 ± 1.78ab	68.49 ± 1.75a	63.54 ± 0.96cb
8	67.95 ± 0.80a	65.38 ± 1.04ab	69.43 ± 1.18a

Means ± SE with different letters within a column are significantly different Tukey's test, α = 0.05. Each data point is an average of 4 determination (*n* = 4).

**Table 3:** Changes in total color changes ***(*Δ*E∗)*** and Chroma (C∗) during storage of OPVI and OPVII in PICS-oxy, PICS-noxy and woven bags for 8 month.

	Storage period	OPVI			OPVII		
		PICS-oxy	PICS-noxy	Woven	PICS-oxy	PICS-noxy	Woven
**Total color change *(*Δ*E∗)***	0	0	0	0	0	0	0
	0.5	5.61	3.82	3.06	3.25	2.87	3.17
	2	3.51	1.21	5.15	4.28	1.71	3.41
	4	0.85	3.01	3.08	7.02	7.57	2.38
	8	1.59	6.70	3.08	7.28	4.68	8.70
**Chroma**	0	32.89	32.89	32.89	35.66	35.66	35.66
	0.5	28.18	29.72	35.80	33.27	33.76	36.33
	2	30.11	33.62	32.71	33.29	33.34	35.31
	4	32.44	30.13	31.72	30.13	34.12	36.95
	8	33.46	37.25	34.17	32.87	37.25	34.01

### 3.2 Correlations between carotenoid contents and color parameters

To understand the relationship between carotenoid content and color parameters, coefficients of determination (*R*^*2*^*)* were computed (Table 4). Δ*E∗, h* and *L∗* correlated negatively with all carotenoids regardless of bag type (Table 4). The bigger the Δ*E∗* the higher the probability of detecting the difference in color between samples (Grobelna et al., 2019) and may indicate the magnitude of change of color induced by storage. All storage bags induced total color change greater than 1 (Table 3) which is perceivable by an experienced observer while color change greater than 2 can be detected by consumers (Grobelna et al., 2019) and may suggest loss of quality. The *a∗* and *b∗* values had both positive and negative correlations with carotenoids depending on genotype and storage bag (Table 4). High and significant correlations between carotenoid content and color parameters suggest that color parameters can potentially be used to estimate carotenoid content in biofortified maize grains. Use of color parameters to determine carotenoid losses has been reported in literature (Onwude et al., 2017; Arias et al., 2002; Melendez-Martinez et al., 2003; Andreu-Sevilla et al., 2008; Ruiz et al., 2008; Itle and Kabelka, 2009; Amenya and Wilson, 1997) with different parameter combinations providing good fit depending on species of carotenoids and food type under study. Both linear (Ahmed et al., 2002) and non-linear Onwude et al. (2017) (Arias et al., 2002) relationships have been reported between carotenoid content and color parameters in potato puree. The established relationships between color parameters and carotenoid content in various foods have resulted in development of models that could be used to estimate carotenoid content in those foods.

**Table 4:** Coefficients of determination (*R*^*2*^*)* between carotenoids content and color parameters after 8 month storage in PICS-oxy, PICS-noxy and woven bags for OPVII.

Carotenoids	Storage system	Color parameters				
		*h*	Δ*E∗*	*L∗*	*a∗*	*b∗*
Provitamin A	PICS-oxy	-0.881^#^	-0.887^#^	-0.910^#^	0.855	0.540
	PICS-noxy	-0.951^#^	-0.828	-0.771	0.788	-0.468
	Woven	-0.894	-0.812	-0.774	0.809	0.028
Lutein	PICS-oxy	-0.894^#^	-0.857	-0.920^#^	0.816	0.443
	PICS-noxy	-0.895^#^	-0.704	-0.650	0.655	-0.589
	Woven	-0.898^#^	-0.889^#^	-0.881^#^	0.916^#^	0.242
Zeaxanthin	PICS-oxy	-0.827	-0.782	-0.909^#^	0.699	0.288
	PICS-noxy	-0.762	-0.501	0.428	0.468	-0.634
	Woven	-0.920^#^	-0.868	-0.842	0.924^#^	0.210
All*-trans-β*-carotene	PICS-oxy	-0.830	-0.905	-0.876	0.880	0.656
	PICS-noxy	-0.987^##^	-0.902	-0.840	0.882	-0.319
	Woven	-0.799	-0.730	-0.675	0.715	-0.113
*β*-cryptoxanthin	PICS-oxy	-0.868	-0.827	-0.879	0.798	0.435
	PICS-noxy	-0.939#	-0.817	-0.760	0.775	-0.484
	Woven	-0.853	-0.809	-0.772	0.807	0.027
Total carotenoids	PICS-oxy	-0.864	-0.846	-0.925^#^	0.781	0.408
	PICS-noxy	-0.863	-0.651	-0.585	0.612	-0.586
	Woven	-0.912^#^	-0.867	-0.837	0.897^#^	0.153

Significant levels for each *R*^*2*^ is indicated by number of hatch, #p < 0.05, ##p < 0.01. *h* hue, **Δ***E∗* total color change, *L∗* value, *a∗* value, *b∗* value.

### 3.3 Predictive equations for carotenoid content in maize grain

Different carotenoids had high coefficients of determination with different color parameters depending on type of storage bags (Table 4). Though there were high coefficients of determination between carotenoid content and *h,* Δ*E∗* and *a∗*, there was no significant correlations between carotenoids and color parameters for OPVI (data not shown). However, there were significant coefficients of determination (p < 0.05) between carotenoids and color parameters for OPVII (Table 4). Therefore, prediction equations were derived from color parameters from OPVII. Based on results presented in Table 5 the main predictor for lutein content in all bags were *h (R*^2^ = 0.8073, p = 0.0382) and *a∗* (*R*^2^ = 0.8394, p = 0.0288). When we modeled for both *a∗* and *h* for lutein, the equation became *lutein* = 29.009 + 1.05*a∗* - 0.431*h***,** *R*^2^ increased to 0.8578 but p-value was insignificant (p = 0.1422) (Table 4). This means that *h* and *a∗* explained 81% and 84% of variations in lutein content, respectively. Zeaxanthin was better predicted by *L∗* value (*R*^2^ = 0.8273, p = 0.0322), *h* (*R*^2^ = 0.8465, p = 0.0268) and *a*∗ (*R*^2^ = 0.8536, p = 0.0249), respectively. Similarly, when we modeled for both *h* and *L∗* value, the equation became *zeaxanthin* = 250.81–4.75*h* + 1.78*L∗,* the *R*^2^ increased to 0.8977 but, p-value was insignificant (p = 0.1023). When we modeled for both *a∗* and *h*, *R*^2^ increased but the p-value was not significant. This means that *L∗, a∗, h, h + L∗* and *a∗+ h* explains 83%, 85%, 85%, 90% and 88% of total variation in zeaxanthin content, respectively. Ninety seven percent (97%) and 88% of variation in *β-*carotene and *β*-cryptoxanthin, respectively, were explained by *h* while *L∗* value explained 93% of variation in *cis-β*-carotene. We also found that Δ*E∗* is a good predictor of β-carotene consistent with results in cashew juice and *β-*carotene enriched nanoemulsion (Zepka et al., 2009; Qian et al., 2012). *h* explained 90% of total variation in total provitamin A carotenoids while *L∗* value explained 86% of variations in total carotenoid content of stored grains. Including both *a∗* and *h* in the equation for total carotenoids improved *R*^2^ to 0.8522 but decreased p-value (p = 0.1478). Consistently, *h*∗ gave high and significant *R*^2^ across all the carotenoids (Table 5). The higher *R*^2^ obtained in this study shows that certain color parameters could be used to estimate carotenoids content in maize. Use of predictive models to estimate carotenoid content in grains has an advantage of not requiring expensive equipment and highly trained personnel that characterizes use of LC methods. The lack of one predictor for carotenoids confirms the diversity of carotenoids in maize grains. Our results also indicate that Δ*E∗* is not a good predictor of total carotenoid content in biofortified maize grains indicated by non-significant p-values in almost all carotenoids (data not shown) while *L∗, h∗* and *a∗* can be used to estimate a number of carotenoids in these grains. This means that when using color parameter to estimate individual carotenoid content in maize grains, it is important to have a prior knowledge and understand which parameters correlate or give more information about the carotenoids species of interest. The equation generated in this study could be helpful in estimating carotenoid content in maize grains in resource poor households of developing countries.

**Table 5:** Predictive equations for estimation of carotenoid content in biofortified maize.

Carotenoid	Main Predictor	Equation	*R*^*2*^	p-value
Lutein	*H*	88.39 - 1.10*h*	0.8073	0.0382
	*a∗*	-8.71 + 1.64a∗	0.8394	0.0288
	*a∗+h*	29.01 + 1.05a∗ - 0.43h	0.8578	0.1422
Zeaxanthin	*L∗*	119.45 - 1.54*L∗*	0.8273	0.0322
	*h + L∗*	250.81 - 4.75*h* + 1.78*L∗*	0.8977	0.1023
	*H*	168.81 - 2.05*h*	0.8465	0.0268
	*a∗*	-13.59–3.042*a*∗	0.8536	0.0249
	*a∗+ h*	75.10 + 1.66a∗ + 1.013*h*	0.8836	0.1164
*All trans-β*-carotene	Δ*E∗*	3.55 - 0.29Δ*E∗*	0.8196	0.0345
	*H*	41.22–0.54*h*	0.9737	0.0016
	*A*	-5.94 + 0.77*a∗*	0.7781	0.0477
*cis-β*-carotene	*L∗*	30.445 - 0.43*L∗*	0.9321	0.0077
	*H*	31.5533 - 0.40*h*	0.8785	0.0187
*β-*cryptoxanthin	*L∗*	18.99 - 0.26*L∗*	0.7726	0.0496
	*H*	27.97 - 0.36*h*	0.8810	0.0181
	Δ*E∗*	2.39 - 0.16Δ*E∗*	0.6544	0.0973
Provitamin A carotenoids	*H*	113.02 - 1.46*h*	0.9000	0.0130
Total Carotenoids	*L∗*	261.31 - 3.46*L∗*	0.8567	0.0241
	*h + L∗*	526.53 - 9.23*h* + 2.85*L∗*	0.9190	0.0637
	*H*	349.09 - 4.30*h*	0.8317	0.0309
	*a∗*	-31.12 + 6.236*a*∗	0.8056	0.0388
	*a∗+h*	202.23 + 2.604*a*∗ + 2.66*h*	0.8522	0.1478

*cis-β*-carotene (total of all trans-*β*-carotene isomers calculated as sum of 15*-cis- β*-carotene, 13*-cis-β*-carotene and 9*-cis-β*-carotene). Provitamin A carotenoid is the sum of all-*trans-β*-carotene, *cis-β*-carotene and *β*-cryptoxanthin. Only models with high R^2^ are shown in the table.

## 4 Conclusion

Color change is indicative of carotenoid loss in carotenoid-rich foods. There is no one parameter that can predict all carotenoid species with same precision, therefore, use of the appropriate color parameters would be ideal in order to get the best estimate. In this study we found that total color change (**Δ*E∗)*** alone or in combination with other parameters is a poor estimator of carotenoids in biofortified maize grains. However, other color parameters such as *L∗*, *h* and *a*∗ provide better estimation of various carotenoid species therefore should be considered for use in estimating carotenoid content of biofortified maize grains in developing countries targeted for biofortification but do not have capacity to use LC techniques.

## Declarations

### Author contribution statement

Smith G. Nkhata: Conceived and designed the experiments; Performed the experiments; Analyzed and interpreted the data; Wrote the paper.

### Funding statement

This work was supported by the United States Agency for International Development, as part of the Feed the Future Initiative, under the 10.13039/501100015815CGIAR Fund, and the predecessor fund the Food Security and Crisis Mitigation II grant (EEM-G-00-04-00013) (BHEARD Scholarship).

### Competing interest statement

The authors declare no conflict of interest.

### Additional information

No additional information is available for this paper.
